# Higher severity of infant RSV infections is associated with lower parental quality of life – a European observational study

**DOI:** 10.1186/s41687-026-01157-3

**Published:** 2026-07-24

**Authors:** Ilona Trautmannsberger, Sabina Bösl, Ina Adamek, Christian Apfelbacher, Philippe Beutels, Brigitte Essers, Ulrike Ravens-Sieberer, Catherine Weil-Olivier, Raffaella Nenna, Martin Wetzke, Silke Mader, Christina Tischer, Luc J. I. Zimmermann, the ResQ Family study group, Kajsa Bohlin, Louis Bont, Daniele De Luca, Katarina Eglin, Susanna Esposito, Fabio Midulla, Raffaella Nenna, Barbara Plagg, Audrey Reynaud, Karl Rombo, Sven Arne Silfverdal, Catherine Weil-Olivier, Sven Wellmann, Martin Wetzke, Christian Apfelbacher, Philippe Beutels, Brigitte Essers, Ulrike Ravens-Sieberer

**Affiliations:** 1 Global Foundation for the Care of Newborn Infants (GFCNI), Hofmannstraße 7a, 81378 Munich, Germany; 2 Department of Paediatrics, Research School for Oncology and Reproduction, Maastricht UMC+, P.O. Box 616, Maastricht, 6200 MD The Netherlands; 3 Institute of Social Medicine and Health Systems Research (ISMHSR), Otto von Guericke University Magdeburg, Leipziger Str. 44, 39120 Magdeburg, Germany; 4 Centre for Health Economics Research & Modelling Infectious Diseases (CHERMID), Vaccine & Infectious Disease Institute, Faculty of Medicine and Health Sciences, University of Antwerp, Campus Drie Eiken - Gebouw S, S241, Universiteitsplein 1, Antwerpen, 2610 Belgium; 5 Department of Clinical Epidemiology and Medical Technology Assessment, Maastricht University Medical Centre, PO Box 5800, Maastricht, 6202 AZ The Netherlands; 6 Department of Child and Adolescent Psychiatry, Psychotherapy, and Psychosomatics, University Medical Centre Hamburg-Eppendorf, Martinistraße 52, 20246 Hamburg, Germany; 7 Pediatrics, University of Paris, Paris, France; 8 Department of Maternal, Infantile, and Urological Sciences, Sapienza University of Rome, 00161 Rome, Italy; 9 Clinic for Pediatric Pneumology, Allergology and Neonatology, Hannover Medical School (MHH), Carl-Neuberg-Str. 1, D-30625 Hannover, Germany; 10 Department of Neonatology, Karolinska University Hospital/Karolinska Institutet, Stockholm, Sweden; 11 Department of General Pediatrics and Pediatric Infectious Diseases, Wilhelmina Children’s Hospital, University Medical Center, Utrecht, The Netherlands; 12 Division of Pediatrics and Neonatal Critical Care, A. Béclère Medical Center, Paris Saclay University Hospitals, APHP, Paris, France; 13 Bundesverband Frühgeborene e.V., Frankfurt (Main), Germany; 14 Paediatric Clinic, Pietro Barilla Children’s Hospital, University of Parma, Parma, Italy; 15 Department of Maternal Infantile and Urological Sciences, Sapienza University of Rome, Rome, Italy; 16 Institute of General Practice and Public Health, Provincial College for Health Professions Claudiana, Bolzano, Italy; 17 SOS Préma, Neuilly sur-Seine, France; 18 Riksförbundet Svenska Prematurförbundet, Stockholm, Sweden; 19 Department of Clinical Sciences, Umeå University, Umeå, Sweden; 20 Pediatrics, University of Paris, Paris, France; 21 Department of Neonatology, University Children’s Hospital of Regensburg (KUNO), Hospital St. Hedwig of the Order of St. John, University of Regensburg, Regensburg, Germany; 22 Clinic for Pediatric Pneumology, Allergology and Neonatology, Hannover Medical School (MHH), Hannover, Germany; 23 Institute of Social Medicine and Health Systems Research (ISMHSR), Otto von Guericke University Magdeburg, Magdeburg, Germany; 24 Faculty of Medicine and Health Sciences, University of Antwerp, Antwerp, Belgium; 25 Department of Clinical Epidemiology and Medical Technology Assessment, Maastricht University Medical Centre, Maastricht, The Netherlands; 26 Department of Child and Adolescent Psychiatry, Psychotherapy, and Psychosomatics, University Medical Centre Hamburg-Eppendorf, Hamburg, Germany

**Keywords:** Respiratory syncytial virus, RSV, Quality of life, HRQoL, Family

## Abstract

**Background:**

RSV infections in children affect the entire family. Nevertheless, evidence on the overall impact is scarce. This multi-country observational study aims to investigate the association between disease severity and health-related quality of life (HRQoL) in affected families and to explore the role of factors related to mental well-being.

**Methodology:**

Data were collected during the 2022–2023 RSV season in Germany, France, Italy and Sweden via an online questionnaire from parents of children (24 months) hospitalised for RSV. Parental HRQoL was assessed using the validated caregiver-reported PedsQL Family Impact Module. Exploratory analyses and multivariate linear regression models were performed focusing on potential key determinants.

**Results:**

A total set of 138 participants was assembled. Parents of children with a severe RSV infection had on average − 12.1 [95% CI: −21.2 to − 3.03; *P* = 0.010] lower HRQoL Summary Score than parents of children with low severity index scores. Subsequent stratified analyses revealed a stronger association among parents who had not been offered (adequate) mental health support.

**Conclusion:**

Higher RSV disease severity was associated with lower parental HRQoL. These findings highlight the importance of preventing severe RSV infection and the need for further research to identify and evaluate appropriate (psychosocial) support for affected families.

**Trial registration:**

ClinicalTrials.gov, identifier, NCT05550545. Registered 12 September 2022.

**Supplementary Information:**

The online version contains supplementary material available at 10.1186/s41687-026-01157-3.

## Introduction

The respiratory syncytial virus (RSV) is one of the most common pathogens affecting respiratory health in early childhood [1]. It usually manifests by cold-like symptoms; however, some affected infants develop lower respiratory tract infections (LRTI), leading to acute bronchiolitis or pneumonia [2, 3]. Globally, RSV-associated LRTIs in children under five years of age result in over three million hospital admissions annually, placing a significant burden not only on affected children but also on healthcare systems and society [4, 5].

The impact of an RSV infection is not limited to the acute and directly visible effects of the disease but may also manifest in long-term physical complications (e.g. wheezing, asthma or impaired lung function later in childhood) and psychosocial challenges for the entire family [2, 6–8]. Especially parents are impacted in myriad ways when family life suddenly changes due to an infant’s hospitalisation [9].

A construct that has been successfully used to capture the scope of these implications is the health-related quality of life (HRQoL) concept. HRQoL is acknowledged as a key health outcome indicator in research studies and describes both objective and subjective well-being relative to one’s health or disease status including several dimensions such as physical, emotional, cognitive and mental health aspects [10, 11]. To measure a person’s HRQoL, patient-reported outcome measures (PROMs) are increasingly used, complementing the traditional clinical ways of quantifying health [12].

However, evidence on caregiver-specific PROMs specifically tailored to measure the HRQoL impact on caregivers following severe RSV infection in infants remains limited [8, 13–15]. Factors such as stress, loneliness, and individual coping abilities may influence HRQoL, underscoring the importance of identifying both stressors and preventive factors to support affected families [16, 17].

To address these gaps with a caregiver-centred perspective, the ResQ Family study (ResQ Family: Impact of Respiratory Syncytial Virus hospitalisation on Quality of life of Families – a multi-country study) was initiated by the European Foundation for the Care of Newborn Infants (EFCNI; now Global Foundation for the Care of Newborn Infants, GFCNI). The primary objective of this multi-country observational study was to examine the association between the severity of infant RSV infection requiring hospitalisation and parental health-related quality of life (HRQoL) during the acute phase and at follow-up, measured using a validated caregiver-specific patient-reported outcome instrument. We hypothesised that higher RSV disease severity would be associated with lower parental HRQoL. Secondary objectives were to explore whether factors related to (parental mental health) support and health literacy may modify this association.

## Methods

### Study design

This multi-country observational study was conducted during the 2022–2023 RSV season in Germany, France, Italy and Sweden. Between September 2022 and May 2023, parents and caregivers^1^ of children ($\:\le\:$ 24 months of age) hospitalised for RSV were approached during or shortly after hospitalisation to share their experiences via an online, web-based, open-link questionnaire. Full details of the methodology of the study are provided in a published study protocol [18].

### Involvement of relevant stakeholders

In collaboration with relevant stakeholders, researchers in the scientific affairs and research department of EFCNI developed the framework of the study. To enable a participative research process, a Project Expert Group (PEG) including both healthcare professionals (HCPs) and (affected) parent and patient representatives of all four participating countries was established and involved throughout the project. Researchers specialised in the field of RSV and/or quality of life complemented the multi- and transdisciplinary working group as an External Scientific Advisory Board (ESAB).

### Recruitment of participants

Participants were primarily recruited via social media outreach by EFCNI, supported by its network of national parent organisations as well as partnering HCPs and healthcare societies, who distributed materials both in hospitals and online.

Recruitment occurred within four weeks of the child’s RSV-related hospitalisation (t0). Upon completion of the initial questionnaire, parents were invited via e-mail to participate in a follow-up survey (t1), which was initiated approximately (approx.) six weeks after t0. The median time between t0 and t1 was 44 days (mean 59 days), reflecting variability due to reminder-based recruitment.

### Questionnaire development

Both online questionnaires (initial survey (t0) and follow-up survey (t1)) included a mixture of validated and self-developed items. A total set of 84 single- and multiple-choice closed and one open question for t0 and 23 single- and multiple-choice closed and one open question for t1 were provided to the participants. Data were collected on the (sociodemographic) background and RSV- as well as quality of life-related topics pertaining to both the participating parent and the affected infant. Non-standard items were developed in collaboration with the working group (PEG and ESAB) and cognitively piloted for clarity, relevance, and cross-cultural comprehensibility prior to field implementation, as described in detail in the corresponding study protocol [18]. The master version of the overall survey in English language is shown in a previous publication of the ResQ Family project [19].

#### Severity of the RSV infection

Within the initial questionnaire, parents were asked to complete questions about their child’s respiratory health status. Based on previous literature, a composite severity index was built to estimate the severity of the child’s RSV infection, with both the selected indicators and their weighting informed by previously published severity measures [13, 20–24]. Infants were assigned a score of 1 for each indicator of severity including a hospital stay of $\:\ge\:$ 5 days, a need for supplemental oxygen, a need for apnoea monitoring and confirmed apnoea episodes during hospitalisation and a score of 2 for ICU admission as well as for the need of mechanical ventilation (invasive or non-invasive respiratory support). The scores were then accumulated (range 0–8), with higher scores indicating a more severe course of the infection. A variable classifying the affected children into two groups (≥ 3 points = High Severity Index Score, < 3 points Low Severity Index Score) was used as the main exposure variable to assess disease severity. The cut-off value was chosen to distinguish children with a moderate-to-severe clinical course, characterised by the presence of multiple severity indicators and/or intensive care treatment, from those with milder disease. This categorisation aims to reflect clinically meaningful differences in disease burden [13, 21, 22].

#### Parental health-related quality of life

The Pediatrics Quality of Life Family Impact Module 2.0 (PedsQL FIM) was used as the main parent-reported outcome measurement instrument in the initial as well as in the follow-up survey. The PedsQL FIM is a standardised and caregiver-specific, self-report questionnaire designed to measure the impact of paediatric acute and chronic health conditions on parents’ HRQoL and family functioning with a recall period of one month [25–27]. It consists of 36 Likert-scaled items standing for several dimensions (physical, emotional, social and cognitive functioning, communication, worry, family daily activities and family relationships) generating three sum scores ranging from 0 to 100: the Total Score, the Parent HRQoL Summary Score, and the Family Functioning Summary Score [25]. Higher scores indicate better parental HRQoL and/or family functioning. Validation of the questionnaire was provided in all official languages of the four participating countries (Swedish, German, French, Italian) [26].

In addition to the PedsQL FIM, information on parental health literacy and (mental health) support was collected as potential key factors that may modify the association between RSV disease severity and HRQoL.

### Statistical analysis

The distribution of categorical variables in the study population was presented as absolute numbers and percentages, while continuous variables were described using medians and interquartile ranges. Subgroup analysis aimed to further investigate the relationship between the severity index score group of the child’s infection and the respective covariates. Pearson’s Chi-squared/Fisher’s exact test and ANOVA/Kruskal-Wallis test were applied to examine potential differences between the subcategories.

To assess the association between the severity of the child’s RSV infection and parents’ HRQoL and family functioning, multivariate linear regression models were used. Adjustments to control for possible confounding factors were made a priori for the age, educational level and smoking status of the parent, for the current and gestational age of the child, for the total number of people living in the child’s household and for the participants’ country of origin.

Models were conducted for the overall sample and then stratified by factors related to health literacy and (mental health) support. A change over the observation period (t0 until t1) in all PedsQL FIM sum scores including each separate dimension was calculated applying paired t-tests and lastly, multivariate linear regression models were repeated with the follow-up score results.

Complete case analysis was performed for the main analyses. To assess the robustness of the findings, a sensitivity analysis applying multiple imputation by chained equations under the assumption that data were missing at random was conducted for missing values of exposure, outcomes, and covariates included in the models. All evaluations were performed using the statistical software R (R version 4.2.2; R Core Team, 2023. R: A language and environment for statistical computing. R Foundation for Statistical Computing, Vienna, Austria. https://www.R-project.org/). P-values below 0.05 were considered statistically significant.

## Results

### Study population characteristics

In total, 138 participants with complete information for t0 and 59 for t1 were included in the study. A flowchart of participant inclusion (see Supplement, Figure S1) as well as the distribution of all variables analysed for the total sample and subdivided by country are presented in detail in a previous publication of the ResQ Family project [19]. In short, key baseline characteristics can be found in Table 1, stratified by RSV symptom severity status.

**Table 1:** Baseline characteristics (*n* = 138) overall and by severity index score group

	All participants	Severity index group of affected children		*p*-value^a^
		High	Low	
***Socio-demographic data***				
**Country of origin**	**n = 138**	**n = 112**	**n = 26**	0.7197
France	60	47	13	
Germany	32	25	7	
Italy	28	24	4	
Sweden	18	16	2	
**Sex caregiver**,** n (%)**	**n = 137**	**n = 112**	**n = 25**	0.1602
Male	8 (5.8)	5 (4.5)	3 (12.0)	
Female	129 (94.2)	107 (95.5)	22 (88.0)	
**Age caregiver**	**n = 126**	**n = 104**	**n = 22**	0.345
in years, median (range)	32.5 (22–45)	33 (22–45)	31.5 (26–42)	
**Caregiver education**,** n (%)**	**n = 137**	**n = 112**	**n = 25**	0.2933
Never attended school / no graduation / middle school	3 (2.2)	2 (1.8)	1 (4.0)	
High School / Secondary school certificate	16 (11.7)	12 (10.7)	4 (16.0)	
College	39 (28.5)	35 (31.3)	4 (16.0)	
University degree	79 (57.7)	63 (56.3)	16 (64.0)	
**Age child**	**n = 134**	**n = 110**	**n = 24**	0.898
in months, median (range)	3 (0–28)	3 (0–28)	3.5 (0–27)	
**Sex child**	**n = 138**	**n = 112**	**n = 26**	0.1427
Male	77 (55.8)	66 (58.9)	11 (42.3)	
Female	59 (42.8)	45 (40.2)	14 (53.8)	
Not specified	2 (1.4)	1 (0.9)	1 (3.8)	
**Gestational age**,** n (%)**	**n = 138**	**n = 112**	**n = 26**	0.4653
Extremely preterm: less than 28 weeks	6 (4.3)	6 (5.4)	0 (0.0)	
Very preterm: 28 – < 32 weeks	20 (14.5)	15 (13.4)	5 (19.2)	
Moderate to late preterm: 32 – < 37 weeks	28 (20.3)	21 (18.8)	7 (26.9)	
Mature born: ≥ 37 weeks	84 (60.9)	70 (62.5)	14 (53.8)	
**Active smoker in the household**	**n = 138**	**n = 112**	**n = 26**	0.3163
No	121 (87.7)	100 (89.3)	21 (80.8)	
Yes	17 (12.3)	12 (10.7)	5 (19.2)	
**Number of people living in the same household**	**n = 137**	**n = 111**	**n = 26**	0.0768
median (range)	4 (2–7)	4 (2–7)	4 (3–7)	

Note: Categorical variables were illustrated using absolute numbers and percentages, while continuous variables were described with medians and interquartile ranges; Percentages may not total 100% due to rounding; Significant values (marked in bold) were defined as *p* < 0.05

With the aim of maximising study participation, three parents with an extended recall period (98, 245 and 407 days) as well as caregivers of affected children exceeding 24 months of age (< 27 months) were included in the analyses, however, sensitivity analysis excluding the respective respondents revealed no major changes in the overall results

^a^ Pearson’s Chi-square/ Fisher’s exact test for categorical variables and ANOVA test (or Kruskal-Wallis test if normal distribution was not given) for continuous variables were used to examine potential differences between the groups

### Association between severity of RSV infection in children and parents’ HRQoL and family functioning during the acute infection phase

The overall median score of the severity index was 5.0 (range: 1–8) classifying the study group into 26 patients with a low and 112 patients with a high severity index score, respectively. Baseline characteristics subdivided by the severity of the RSV infection showed no significant differences between the two groups (see Table 1). Average PedsQL FIM scores for the overall sample at time t0 describing parental HRQoL and family functioning during the acute infection phase were 55.6 for the Total Score, 52.5 for the Parent HRQoL Summary Score and 59.4 for the Family Functioning Summary Score. A subdivision by the severity of the RSV infection revealed differences between the groups with a significantly lower Total Score and Parent HRQoL Summary Score for parents with children exhibiting a high severity index score (*p* = 0.0118 and *p* = 0.0053; for PedsQL FIM scores broken down by dimension see Table 2).

**Table 2:** PedsQL FIM scores at t0 (*n* = 138) overall and by severity group

	All participants	Severity index group of affected children		*p*-value^a^
		High	Low	
**PedsQL FIM Sum Scores** ^b^				
**Total Score**	**n = 137**	**n = 112**	**n = 25**	**0.0118**
Median (range)	55.56 (6.25–94.44)	54.17 (6.25–90.28)	63.89 (36.11–94.44)	
**Parent HRQoL Summary Score**	**n = 137**	**n = 112**	**n = 25**	**0.0053**
Median (range)	52.5 (5.00–95.00)	56 (5-88.75)	65 (26.25-95)	
**Family functioning Summary Score**	**n = 135**	**n = 110**	**n = 25**	0.105
Median (range)	59.38 (3.16–100.00)	59.38 (3.12–100)	65.62 (43.75–100)	
**PedsQL FIM individual dimensions**				
*Physical functioning score*	**n = 137**	**n = 112**	**n = 25**	**0.0153**
Median (range)	41.67 (0.00-91.67)	39.58 (0-83.33)	54.17 (16.67–91.67)	
*Emotional functioning score*	**n = 137**	**n = 112**	**n = 25**	0.0868
Median (range)	50.0 (0.00–95.00)	47.5 (0–95)	65 (0–90)	
*Social functioning score*	**n = 137**	**n = 112**	**n = 25**	**0.0274**
Median (range)	50.00 (6.25–100.0)	50 (6.35–100)	62.5 (25–100)	
*Cognitive functioning score*	**n = 137**	**n = 112**	**n = 25**	**0.0102**
Median (range)	60.0 (0.00-100.00)	55 (0-100)	75 (30–100)	
*Communication score*	**n = 137**	**n = 112**	**n = 25**	**0.491**
Median (range)	66.67 (8.33–100.00)	66.67 (8.33–100)	75 (25–100)	
*Worry score*	**n = 137**	**n = 112**	**n = 25**	0.174
Median (range)	60.0 (0.00-100.00)	60 (0-100)	65 (15–96)	
*Daily activities score*	**n = 136**	**n = 111**	**n = 25**	0.52
Median (range)	33.33 (0.00-100.00)	33.33 (0-100)	41.67 (0-100)	
*Family relationsships score*	**n = 136**	**n = 111**	**n = 25**	0.334
Median (range)	72.5 (0.00-100.00)	70 (0-100)	75 (40–100)	

^a^ ANOVA test (or Kruskal-Wallis test if normal distribution was not given) for continuous variables were used to examine potential differences between the groups

^b^ The PedsQL FIM generated three sum scores ranging from 0-100 (with 100 being the best possible score): the Total Score (including all dimensions: physical, emotional, social and cognitive functioning, communication, worry, family daily activities and family relationships), the Parent HRQoL Summary Score (including physical, emotional, social and cognitive functioning), and the Family Functioning Summary Score (including daily activities and family relationships) [25]

Multivariate analysis revealed that the severity of a child’s RSV infection significantly impacted parents’ HRQoL as well as family functioning. In detail, results showed that parents of children with a severe RSV infection (high severity index score) had on average − 10.3 [95% CI: −18.6 to − 1.90; *P* = 0.017] lower Total Score, − 12.1 [95% CI: −21.2 to − 3.03; *P* = 0.010] lower Parental HRQoL Summary Score and − 10.5 [95% CI: −20.5 to − 0.47; *P* = 0.04] lower Family Functioning Summary Score, respectively, than parents of children assigned to the “Low” severity index score group.

### Potential key factors within the interaction between RSV disease severity and HRQoL

Subsequent stratified analyses related to health literacy and (mental health) support can be found in (Table 3a – e). The impairment of parental health-related quality of life due to a severe RSV infection was particularly evident among parents who were not offered (adequate) mental health support during the child’s hospitalisation. However, no significant association with the Family Functioning Summary Score was observed in either group (Table 3a). Further analysis on parental health literacy related factors indicated a significant correlation for all three sum scores within the group of parents with high health literacy or better knowledge/understanding about RSV and its multifaceted consequences (see Table 3b – e). A detailed overview of all health literacy and mental health support variables included in the stratified analyses is provided in the Supplement (Table S1).

**Table 3:** Association between RSV severity and parental HRQoL and family functioning at t0 stratified by support and health literacy factors

a) Receiving adequate information about mental health support						
	Total		Parent HRQoL		Family Functioning	
	Coef. [95% CI]	p-value	Coef. [95% CI]	p-value	Coef. [95% CI]	p-value
**Overall sample**						
*Low*	Ref.		Ref.		Ref.	
*High*	−10.5 [− 19.0 to − 1.96]	**0.016**	−12.4 [− 21.7 to − 3.00]	**0.010**	-10.3 [-20.7 to 0.166]	0.054
*Adjusted R²*	0.13		0.15		-0.01	
**No offer of (adequate) mental health support**						
*Low*	Ref.		Ref.		Ref.	
*High*	-11.2 [-21.1 to -1.27]	**0.028**	-13.0 [-23.5 to -2.53]	**0.016**	-12.6 [-25.5 to 0.334]	0.056
*Adjusted R²*	0.09		0.13		-0.03	
**Offer of (adequate) mental health support**						
*Low*	Ref.		Ref.		Ref.	
*High*	-12.6 [-45.5 to 20.3]	0.424	-11.4 [-50.8 to 28.0]	0.54	-6.59 [-33.9 to 20.7]	0.612
*Adjusted R²*	0.04		0.01		0.17	
Models were adjusted for the age, educational level and smoking status of the caregiver, for the current and gestational age of the child, for the total number of people living in the child’s household, the participants’ country of origin and the presence of (adequate) mental health support. Models stratified for the receipt of (adequate) mental health support were adjusted for the same variables except for mental health support						
Abbreviations: CI, confidence interval; Coef., regression coefficient; HRQoL, health-related quality of life; Ref., reference						

b) Knowledge where to find support for managing mental health problems						
	Total	Parent HRQoL	Family Functioning			
	Coef. [95% CI]	p-value	Coef. [95% CI]	p-value	Coef. [95% CI]	p-value
**Overall sample**						
*Low*	Ref.		Ref.		Ref.	
*High*	−12.5 [− 20.9 to − 4.05]	**0.004**	−14.6 [− 23.9 to − 5.38]	**0.002**	-11.2 [-21.7 to -0.652]	**0.038**
*Adjusted R²*	0.25		0.27		0.03	
**No knowledge about mental health support**						
*Low*	Ref.		Ref.		Ref.	
*High*	-2.75 [-16.2 to -10.7]	0.668	-3.43 [-20.5 to 13.6]	0.673	-2.16 [-26.5 to 22.2]	0.825
*Adjusted R²*	0.13		0.002		-0.03	
**Knowledge about mental health support**						
*Low*	Ref.		Ref.		Ref.	
*High*	-19.1 [-30.4 to -7.81]	**0.001**	-21.8 [-34.0 to -9.72]	**< 0.001**	-19.7 [-31.9.8 to -7.49]	**0.002**
*Adjusted R²*	0.22		0.26		0.07	
Models were adjusted for the age, educational level and smoking status of the caregiver, for the current and gestational age of the child, for the total number of people living in the child’s household, the participants’ country of origin and the participants’ knowledge where to find support for managing mental health problems. Models stratified for the knowledge where to find support for managing mental health problems were adjusted for the same variables except for the knowledge about mental health support						
Abbreviations: CI, confidence interval; Coef., regression coefficient; HRQoL, health-related quality of life; Ref., reference						

c) Awareness of RSV and its possible complications/ consequences for the child						
	Total	Parent HRQoL	Family Functioning			
	Coef. [95% CI]	p-value	Coef. [95% CI]	p-value	Coef. [95% CI]	p-value
**Overall sample**						
*Low*	Ref.		Ref.		Ref.	
*High*	−10.5 [− 19.0 to − 1.94]	**0.017**	−12.1 [− 21.4 to − 2.84]	**0.011**	-11.2 [-21.3 to -1.06]	**0.031**
*Adjusted R²*	0.12		0.15		-0.02	
**No awareness**						
*Low*	Ref.		Ref.		Ref.	
*High*	3.47 [-28.8 to -35.7]	0.815	6.58 [-26.2 to 39.4]	0.664	-12.0 [-49.9 to 25.9]	0.49
*Adjusted R²*	-0.2		0.03		-0.24	
**Awareness**						
*Low*	Ref.		Ref.		Ref.	
*High*	-12.7 [-22.7 to -2.71]	**0.013**	-15.6 [-26.5 to -4.74]	**0.005**	-12.4 [-24.1 to -0.62]	**0.039**
*Adjusted R²*	0.11		0.13		-0.03	
Models were adjusted for the age, educational level and smoking status of the caregiver, for the current and gestational age of the child, for the total number of people living in the child’s household, the participants’ country of origin and the participants’ awareness of RSV and its possible complications/ consequences for the child. Models stratified for the participants’ awareness of RSV and its possible complications/ consequences for the child were adjusted for the same variables except for awareness						
Abbreviations: CI, confidence interval; Coef., regression coefficient; HRQoL, health-related quality of life; Ref., reference						

d) Awareness of consequences of an RSV infection and hospitalisation on the family						
	Total	Parent HRQoL	Family Functioning			
	Coef. [95% CI]	p-value	Coef. [95% CI]	p-value	Coef. [95% CI]	p-value
**Overall sample**						
*Low*	Ref.		Ref.		Ref.	
*High*	−9.06 [− 17.5 to − 0.65]	**0.035**	−11.0 [− 20.2 to − 1.77]	**0.02**	-9.28 [-19.4 to -0.83]	0.071
*Adjusted R²*	0.17		0.19		-0.02	
**No awareness**						
*Low*	Ref.		Ref.		Ref.	
*High*	3.44 [-11.6 to 18.5]	0.647	2.41 [-13.4 to 18.2]	0.760	3.89 [-14.0.9 to 21.8]	0.66
*Adjusted R²*	0.06		0.13		-0.12	
**Awareness**						
*Low*	Ref.		Ref.		Ref.	
*High*	-15.2 [-25.7 to -4.71]	**0.006**	-18.6 [-31.0 to -6.18]	**0.004**	-15.2 [-28.2 to -2.18]	**0.023**
*Adjusted R²*	0.3		0.26		0.12	
Models were adjusted for the age, educational level and smoking status of the caregiver, for the current and gestational age of the child, for the total number of people living in the child’s household, the participants’ country of origin and the participants’ awareness of consequences of the child’s infection and hospitalisation due to RSV for the family. Models stratified for the participants’ awareness of consequences of the child’s infection and hospitalisation due to RSV for the family were adjusted for the same variables except for awareness						
Abbreviations: CI, confidence interval; Coef., regression coefficient; HRQoL, health-related quality of life; Ref., reference						

e) Awareness of prevention measures						
	Total	Parent HRQoL	Family Functioning			
	Coef. [95% CI]	p-value	Coef. [95% CI]	p-value	Coef. [95% CI]	p-value
**Overall sample**						
*Low*	Ref.		Ref.		Ref.	
*High*	−9.48 [− 17.6 to − 1.31]	**0.024**	−11.4 [− 20.4 to − 2.45]	**0.013**	-9.70 [-19.6 to -0.18]	0.054
*Adjusted R²*	0.18		0.19		0.01	
**No awareness**						
*Low*	Ref.		Ref.		Ref.	
*High*	-15.7 [-36.5 to 5.02]	0.129	-15.5 [-35.4 to 4.44]	0.12	-18.8 [-50.5 to 12.9]	0.228
*Adjusted R²*	0.27		0.41		-0.05	
**Awareness**						
*Low*	Ref.		Ref.		Ref.	
*High*	-11.0 [-21.0 to -0.95]	**0.032**	-13.7 [-25.0 to -2.51]	**0.017**	-12.7 [-24.3 to -1.10]	**0.032**
*Adjusted R²*	0.17		0.16		-0.03	
Models were adjusted for the age, educational level and smoking status of the caregiver, for the current and gestational age of the child, for the total number of people living in the child’s household, the participants’ country of origin and the participants’ awareness of measures for preventing RSV. Models stratified for the participants’ awareness of measures for preventing RSV were adjusted for the same variables except for awareness						
Abbreviations: CI, confidence interval; Coef., regression coefficient; HRQoL, health-related quality of life; Ref., reference						

### Changes in parental HRQoL and family functioning from the acute infection phase (t0) to follow-up (t1)

Compared to the follow-up measurement t1, participants reported significantly lower Total score, Parent HRQoL Summary Score and Family Functioning Summary Score during the acute infection phase (t0). At subscale level, parents of RSV-infected children showed no significant differences for “cognitive functioning,” “communication,” and “family relationships” (see Table S2). In addition, repeated multivariate linear regression models depicted no significant evidence for the association of the severity of the infection and PedsQL FIM scores at t1 (data not shown). Follow-up responders and non-responders did not differ significantly in baseline RSV severity or PedsQL FIM scores.

Findings of the sensitivity analysis were generally consistent with those of the main analyses with regard to both the direction and magnitude of the observed associations (data not shown).

## Discussion

The aim of the current study was to investigate the association between the severity of infant RSV infections with hospitalisation in relation to HRQoL in affected parents of four European countries. Special attention was given to potential key determinants of HRQoL impact. Analyses revealed that particularly in the acute infection phase of a severe RSV case, the HRQoL in involved parents is impaired. Mental health support during the RSV-associated hospital stay and parental health literacy emerged as potentially relevant factors associated with a perceived burden among affected families.

In our study, higher RSV severity was associated with a 12.1-point reduction in parental HRQoL (95% CI: −21.2 to − 3.03, *P* = 0.010). Although no established minimal clinically important difference exists for the PedsQL FIM in this context, the magnitude of the observed difference suggests a substantial impact on parental well-being. This aligns with the general trends reported by Chow et al., who found that the severity of influenza-like illnesses (ILI) in infants aged 6 to 36 months are significantly correlated with parental quality of life (QoL) [28]. Similarly, Overmann et al. reported an association between lower QoL scores and severity of illness in a heterogeneous US population of caregivers of young children (6–48 months) with ILI during the COVID-19 pandemic. Specifically, parents who perceived their child’s illness as very severe had substantially lower scores in the Emotions domain, with values as low as 2.61 compared to 6.00 for those who perceived the illness as mild (*P* = 0.0269), highlighting the emotional toll of perceived illness severity [29]. Peralta and colleagues investigated a different topic and population (respiratory morbidity in preschool and school‑age children born very preterm) but used the same validated caregiver-specific QoL measurement instrument (PedsQL FIM). They also described an increasing deterioration in parental HRQoL with worsening respiratory symptoms in the children [30].

Although these studies investigated different populations and used varied QoL measurement tools, the consistent pattern of declining QoL across various dimensions emphasises the strong association between respiratory illness severity and family well-being. Our study contributes to this body of evidence by quantifying the impact of RSV infection severity on both parental HRQoL and family functioning, showing reductions of 10.3 points (95% CI: −18.6 to − 1.90, *P* = 0.017) in overall QoL scores compared to parents of children with milder RSV. The use of a caregiver-specific QoL measurement tool allowed us a comprehensive understanding of the impact on families, capturing dimensions of well-being that might be overlooked by more general instruments. At follow-up, no significant associations between RSV severity and PedsQL FIM scores were observed. This may partly reflect the overall improvement in parental HRQoL and family functioning observed approx. six weeks after the acute infection phase.

These findings underscore the importance of early intervention and prevention strategies to mitigate the severity of RSV and its broader impacts on family life. New approaches, such as immunisation with long-acting monoclonal antibodies and maternal vaccination, are promising avenues for reducing both the incidence and severity of RSV infections globally [31, 32].

Whilst minimising the impact at population level is likely to have the greatest positive effect on parents’ HRQoL, not all infections can be prevented. We therefore aimed to explore factors that may modify the impact of severe RSV infections on affected patients and their surroundings. The associations between a severe infection and QoL appeared to be stronger in affected families without mental health support services. This supports the hypothesis that psychosocial support offers for parents, such as counselling, self-help or parent groups during the hospitalisation of the child, may be associated with lower individual HRQoL burden among parents with children experiencing severe RSV infection. Similar associations have been found for other diseases such as neurodevelopmental and mental disorders as well as chronic diseases in children [33–35]. However, no significant modification of the association between disease severity and family functioning was observed in relation to mental health support. One possible explanation is that mental health support may primarily address individual well-being, whereas family functioning encompasses broader relational dynamics that may be less immediately responsive to such support.

Additionally, the significant association between disease severity and parental quality of life observed only in the group with higher disease awareness and better knowledge about mental health support suggests that health literacy-related factors may play an important role in how the impact of a child’s RSV infection is perceived and reported. Parents with higher health literacy may have a better understanding of their child’s disease and its potential consequences, potentially influencing their ability to articulate the impact on their own well-being and to access and utilise available resources. At the same time, such heightened awareness of potential complications may also intensify perceived burden during the acute phase of severe illness, thereby strengthening the observable association between disease severity and HRQoL. Conversely, parents with lower health literacy may be less able to recognise or articulate the impact on their quality of life, potentially leading to a less evident association. The observed associations may therefore reflect both differences in the perception and reporting of psychosocial burden and differences in the lived experience of coping with a child’s severe RSV infection. These findings do not diminish the overall importance of health literacy; rather, they highlight its role in enabling affected families to recognise, communicate, and seek support for psychosocial challenges associated with their child’s disease and emphasise its potential relevance within family-centred care approaches [36, 37].

Further research is needed to identify and evaluate the types, timing, and intensity of interventions enhancing health literacy and (psychosocial) support specifically targeted to families affected by RSV.

According to the results of our study, another potentially relevant factor may lie in the presence of good family relationships. Firstly, the correlation between the severity of the infection and the influence on the PedsQL FIM scores is weaker for the Family Functioning Summary Score – which includes the family relationship dimension (communication, conflicts, decision making, problem solving, stress within the family). Secondly, when comparing the two measurement time points t0 and t1, it can be clearly seen that the family relationship dimension remains stable over the observation period and is not significantly impaired during the acute infection phase. This observation suggests that good family relationships may be associated with more favourable parental HRQoL. Even though this association has not been the subject of much evaluation – and particularly not in the context of RSV – current research points in the same direction [38].

### Strengths and limitations

The ResQ Family project is, to our knowledge, the first study incorporating and giving an equal voice to relevant stakeholders, including parents, HCPs, and researchers, which allowed us to identify previously unmet needs as well as potential beneficial factors with regard to the impact of RSV. We have placed a great value on using a validated and caregiver-specific instrument to address HRQoL of parents of affected children and combined it with further items based on existing literature and the continuous exchange with our multi- and transdisciplinary working group [8, 13–15, 25]. This participatory research approach ensured a high level of expertise, thus covering all important aspects and simultaneously ensuring comprehensibility for participants from all educational backgrounds. Thereby, we identified potential prevention and support structures for RSV-affected families and raised awareness among HCPs, patient representatives, decision-makers and the general public to ultimately contribute to efforts to reduce (severe) RSV cases across Europe in the future.

However, some limitations need to be considered. The observational cohort design of the study restricts the analysis to the extent that no causal relationships can be revealed as direct effects cannot be estimated due to the lack of a control group. Nevertheless, compared to a community sample of healthy children (means of the Total Score = 70.8, the Parent HRQoL Summary Score = 69.4 and the Family Functioning Summary Score = 65.5), ResQ Family study participants reported substantially lower sum scores, indicating a negative impact from RSV-related hospitalisations in the current study [27].

As recruitment was conducted pragmatically during a single RSV season across participating countries, no formal a priori sample size calculation was performed. The relatively small sample size for some groups may have diminished the power of the analyses, especially regarding the comparison of baseline characteristics between the two severity score groups and the stratified multivariate models. The smaller follow-up sample may also have reduced statistical power, and potential attrition bias cannot be fully excluded. These limitations also affected the ability to explore inter-country variability, especially for countries with lower participation rates, such as Sweden (*n* = 18). Additionally, the high number of preterm infants in the French cohort may have influenced the observed association between disease severity and HRQoL, though sensitivity analyses excluding children born before 37 weeks of pregnancy showed consistent findings. These country-specific sample characteristics may limit the generalisability of the findings to national contexts and warrant cautious interpretation of cross-country comparisons, particularly given potential differences in healthcare and support systems. Variability in follow-up timing may have further influenced t1 findings, as parental HRQoL may change over the course of recovery following acute disease.

Using social media for participant recruitment may have introduced selection and recall biases due to the voluntary response nature of the procedure and the reliance on parental self-reporting [39, 40]. Recruiting parents during or shortly after hospitalisation might have minimized recall bias, although the one-month recall period of the PedsQL FIM may not have fully aligned with the acute phase of illness for all participants. In addition, participation required digital access and engagement, potentially leading to an overrepresentation of parents with higher educational attainment and health literacy. This may limit the generalisability of the findings, particularly with regard to the observed associations involving health literacy and mental health support and may result in an underrepresentation of the burden experienced by more vulnerable families. However, involving healthcare professionals in outpatient settings likely contributed to a more diverse study sample.

As several variables, including HRQoL, health literacy, and mental health support, were based on parental self-report, common method variance and same-source bias cannot be excluded. Furthermore, the cross-sectional nature of the analyses limits conclusions regarding the direction of observed associations. For example, parents experiencing greater psychosocial burden may be more likely to receive mental health support or to perceive available support as insufficient. However, the primary exposure variable was derived from clinically relevant indicators such as ICU admission, respiratory support, and length of hospital stay, which are less susceptible to subjective interpretation. Although informed by previous RSV studies, the severity index is not externally validated, and alternative score constructions could be considered.

The extension of the originally defined inclusion criteria (age of the child and recall period) may have further influenced the analysis; however, sensitivity analysis revealed no major changes in the direction and magnitude of the results. Lastly, despite adjusting for potential confounders based on existing literature, residual confounding cannot be ruled out.

## Conclusion

The ResQ Family study reveals compelling evidence that particularly during the acute phase of a severe RSV infection, the quality of life of affected families is impaired. It is therefore important to primarily prevent a severe infection from the outset by continuously raising awareness for the disease itself and advancing prevention measures.

The observed associations involving mental health support and parental health literacy highlight areas for future research and may help inform family-centred care approaches during RSV hospitalisations. Targeted psychosocial interventions implemented during the hospital stay should be further explored and evaluated to determine their potential to mitigate parental burden and improve family well-being.

These findings may also carry implications for healthcare settings and policy, including RSV prevention campaigns and efforts to strengthen psychosocial support for affected families. Ultimately, they underscore the importance of valuing, including, and empowering parents as key partners in their child’s care.

## Supplementary Information

Below is the link to the electronic supplementary material.

Supplementary Material 1

## Data Availability

All data generated or analysed during this study are included in this published article and its supplementary information files. The datasets used during the current study are available from the corresponding author on reasonable request.

## Footnotes

1 For simplicity, the term “parents” is used throughout the manuscript and refers to all participating parents and caregivers.
