# Supplementary material for: Higher severity of infant RSV infections is associated with lower parental quality of life – a European observational study
Source: J Patient Rep Outcomes. 2026 Jul 24;10:127. doi: 10.1186/s41687-026-01157-3 (PMC13407400; doi:10.1186/s41687-026-01157-3)
Supplement: Supplementary file 1 — Supplementary Material 1 [file 41687_2026_1157_MOESM1_ESM.docx]

**Supplementary Information**

**Higher severity of infant RSV infections is associated with lower parental quality of life – a European observational study**

# **Figures**

# **
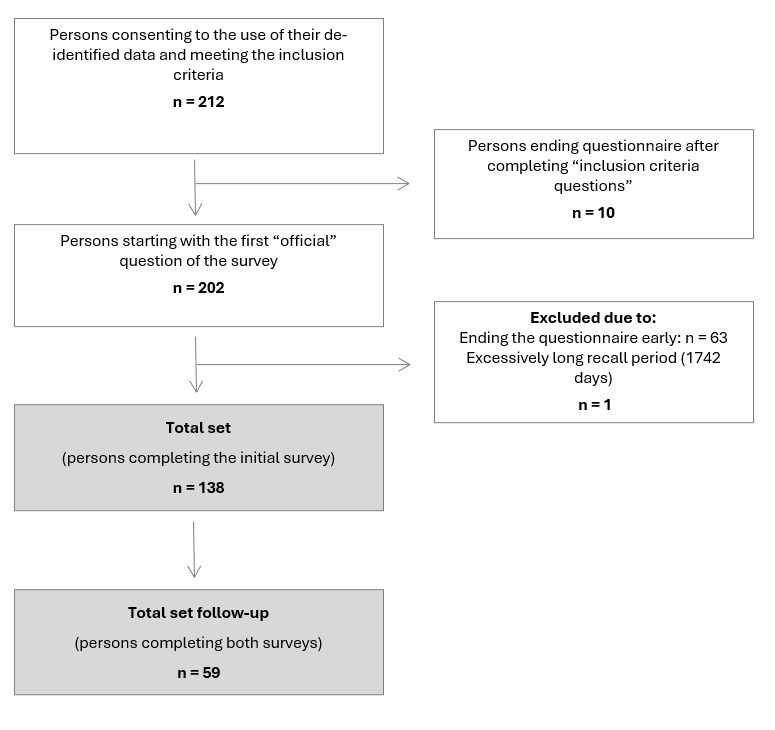
**

# **Figure S1:** Flow chart of questionnaire respondents*

# *The figure has previously been published as part of the ResQ Family project [19] and is reproduced here for completeness.

# **Tables**

**Table S1:** Relationships between severity of RSV infection in children (low versus (vs.) high severity index score) and parents’ HRQoL and family functioning at timepoint t0 stratified by factors related to health literacy and (mental health) support; long version

1. **Receiving adequate information about mental health support**

|  | Total | | Parent HRQoL | | Family Functioning | |
| --- | --- | --- | --- | --- | --- | --- |
|  | **Coef. [95% CI]** | **p-value** | **Coef. [95% CI]** | **p-value** | **Coef. [95% CI]** | **p-value** |
| **Overall sample** |  | | | | | |
| Low | Ref. |  | Ref. |  | Ref. |  |
| High | −10.5 [−19.0 to −1.96] | **0.016** | −12.4 [−21.7 to −3.00] | **0.010** | -10.3 [-20.7 to 0.166] | 0.054 |
| **No offer of (adequate) mental health support** |  |  |  |  |  |  |
| Low | Ref. |  | Ref. |  | Ref. |  |
| High | -11.2 [-21.1 to -1.27] | **0.028** | -13.0 [-23.5 to -2.53] | **0.016** | -12.6 [-25.5 to 0.334] | 0.056 |
| **Offer of (adequate) mental health support** |  |  |  |  |  |  |
| Low | Ref. |  | Ref. |  | Ref. |  |
| High | -12.6 [-45.5 to 20.3] | 0.424 | -11.4 [-50.8 to 28.0] | 0.54 | -6.59 [-33.9 to 20.7] | 0.612 |

Models were adjusted for the age, educational level and smoking status of the caregiver, for the current and gestational age of the child, for the total number of people living in the child’s household, the participants’ country of origin and the presence of (adequate) mental health support. Models stratified for the receipt of (adequate) mental health support were adjusted for the same variables except for mental health support.

Abbreviations: CI, confidence interval; Coef., regression coefficient; HRQoL, health-related quality of life; Ref., reference.

1. **Knowledge where to find support for managing mental health problems**

|  | Total | | Parent HRQoL | | Family Functioning | |
| --- | --- | --- | --- | --- | --- | --- |
|  | **Coef. [95% CI]** | **p-value** | **Coef. [95% CI]** | **p-value** | **Coef. [95% CI]** | **p-value** |
| **Overall sample** |  | | | | | |
| Low | Ref. |  | Ref. |  | Ref. |  |
| High | −12.5 [−20.9 to −4.05] | **0.004** | −14.6 [−23.9 to −5.38] | **0.002** | -11.2 [-21.7 to -0.652] | **0.038** |
| **No knowledge about mental health support** |  |  |  |  |  |  |
| Low | Ref. |  | Ref. |  | Ref. |  |
| High | -2.75 [-16.2 to -10.7] | 0.668 | -3.43 [-20.5 to 13.6] | 0.673 | -2.16 [-26.5 to 22.2] | 0.825 |
| **Knowledge about mental health support** |  |  |  |  |  |  |
| Low | Ref. |  | Ref. |  | Ref. |  |
| High | -19.1 [-30.4 to -7.81] | **0.001** | -21.8 [-34.0 to -9.72] | **<0.001** | -19.7 [-31.9.8 to -7.49] | **0.002** |

Models were adjusted for the age, educational level and smoking status of the caregiver, for the current and gestational age of the child, for the total number of people living in the child’s household, the participants’ country of origin and the participants’ knowledge where to find support for managing mental health problems. Models stratified for the knowledge where to find support for managing mental health problems were adjusted for the same variables except for the knowledge about mental health support.

Abbreviations: CI, confidence interval; Coef., regression coefficient; HRQoL, health-related quality of life; Ref., reference.

1. **Awareness of RSV and its possible complications/ consequences for the child**

|  | Total | | Parent HRQoL | | Family Functioning | |
| --- | --- | --- | --- | --- | --- | --- |
|  | **Coef. [95% CI]** | **p-value** | **Coef. [95% CI]** | **p-value** | **Coef. [95% CI]** | **p-value** |
| **Overall sample** |  | | | | | |
| Low | Ref. |  | Ref. |  | Ref. |  |
| High | −10.5 [−19.0 to −1.94] | **0.017** | −12.1 [−21.4 to −2.84] | **0.011** | -11.2 [-21.3 to -1.06] | **0.031** |
| **No awareness** |  |  |  |  |  |  |
| Low | Ref. |  | Ref. |  | Ref. |  |
| High | 3.47 [-28.8 to -35.7] | 0.815 | 6.58 [-26.2 to 39.4] | 0.664 | -12.0 [-49.9 to 25.9] | 0.49 |
| **Awareness** |  |  |  |  |  |  |
| Low | Ref. |  | Ref. |  | Ref. |  |
| High | -12.7 [-22.7 to -2.71] | **0.013** | -15.6 [-26.5 to -4.74] | **0.005** | -12.4 [-24.1 to -0.62] | **0.039** |

Models were adjusted for the age, educational level and smoking status of the caregiver, for the current and gestational age of the child, for the total number of people living in the child’s household, the participants’ country of origin and the participants’ awareness of RSV and its possible complications/ consequences for the child. Models stratified for the participants’ awareness of RSV and its possible complications/ consequences for the child were adjusted for the same variables except for awareness.

Abbreviations: CI, confidence interval; Coef., regression coefficient; HRQoL, health-related quality of life; Ref., reference.

1. **Awareness of consequences of an RSV infection and hospitalisation on the family**

|  | Total | | Parent HRQoL | | Family Functioning | |
| --- | --- | --- | --- | --- | --- | --- |
|  | **Coef. [95% CI]** | **p-value** | **Coef. [95% CI]** | **p-value** | **Coef. [95% CI]** | **p-value** |
| **Overall sample** |  | | | | | |
| Low | Ref. |  | Ref. |  | Ref. |  |
| High | −9.06 [−17.5 to −0.65] | **0.035** | −11.0 [−20.2 to −1.77] | **0.02** | -9.28 [-19.4 to -0.83] | 0.071 |
| **No awareness** |  |  |  |  |  |  |
| Low | Ref. |  | Ref. |  | Ref. |  |
| High | 3.44 [-11.6 to 18.5] | 0.647 | 2.41 [-13.4 to 18.2] | 0.760 | 3.89 [-14.0.9 to 21.8] | 0.66 |
| **Awareness** |  |  |  |  |  |  |
| Low | Ref. |  | Ref. |  | Ref. |  |
| High | -15.2 [-25.7 to -4.71] | **0.006** | -18.6 [-31.0 to -6.18] | **0.004** | -15.2 [-28.2 to -2.18] | **0.023** |

Models were adjusted for the age, educational level and smoking status of the caregiver, for the current and gestational age of the child, for the total number of people living in the child’s household, the participants’ country of origin and the participants’ awareness of consequences of the child’s infection and hospitalisation due to RSV for the family. Models stratified for the participants’ awareness of consequences of the child’s infection and hospitalisation due to RSV for the family were adjusted for the same variables except for awareness.

Abbreviations: CI, confidence interval; Coef., regression coefficient; HRQoL, health-related quality of life; Ref., reference.

1. **Awareness of prevention measures**

|  | Total | | Parent HRQoL | | Family Functioning | |
| --- | --- | --- | --- | --- | --- | --- |
|  | **Coef. [95% CI]** | **p-value** | **Coef. [95% CI]** | **p-value** | **Coef. [95% CI]** | **p-value** |
| **Overall sample** |  | | | | | |
| Low | Ref. |  | Ref. |  | Ref. |  |
| High | −9.48 [−17.6 to −1.31] | **0.024** | −11.4 [−20.4 to −2.45] | **0.013** | -9.70 [-19.6 to -0.18] | 0.054 |
| **No awareness** |  |  |  |  |  |  |
| Low | Ref. |  | Ref. |  | Ref. |  |
| High | -15.7 [-36.5 to 5.02] | 0.129 | -15.5 [-35.4 to 4.44] | 0.12 | -18.8 [-50.5 to 12.9] | 0.228 |
| **Awareness** |  |  |  |  |  |  |
| Low | Ref. |  | Ref. |  | Ref. |  |
| High | -11.0 [-21.0 to -0.95] | **0.032** | -13.7 [-25.0 to -2.51] | **0.017** | -12.7 [-24.3 to -1.10] | **0.032** |

Models were adjusted for the age, educational level and smoking status of the caregiver, for the current and gestational age of the child, for the total number of people living in the child’s household, the participants’ country of origin and the participants’ awareness of measures for preventing RSV. Models stratified for the participants’ awareness of measures for preventing RSV were adjusted for the same variables except for awareness.

Abbreviations: CI, confidence interval; Coef., regression coefficient; HRQoL, health-related quality of life; Ref., reference.

1. **Understanding HCPs regarding children’s RSV infection and treatment**

|  | Total | | Parent HRQoL | | Family Functioning | |
| --- | --- | --- | --- | --- | --- | --- |
|  | **Coef. [95% CI]** | **p-value** | **Coef. [95% CI]** | **p-value** | **Coef. [95% CI]** | **p-value** |
| **Overall sample** |  | | | | | |
| Low | Ref. |  | Ref. |  | Ref. |  |
| High | −10.7 [−18.9 to −2.54] | **0.011** | −12.6 [−21.5 to −3.78] | **0.006** | -10.9 [-20.8 to -0.99] | **0.031** |
| **Low understanding** |  |  |  |  |  |  |
| Low | Ref. |  | Ref. |  | Ref. |  |
| High | -11.2 [-36.3 to 14.0] | 0.341 | -7.96 [-38.3 to 22.4] | 0.567 | -22.4 [-71.7 to 26.8] | 0.33 |
| **High understanding** |  |  |  |  |  |  |
| Low | Ref. |  | Ref. |  | Ref. |  |
| High | -9.30 [-19.3 to 0.647] | 0.067 | -10.8 [-21.5 to -0.053] | **0.049** | -9.94 [-21.2 to 1.29] | 0.082 |

Models were adjusted for the age, educational level and smoking status of the caregiver, for the current and gestational age of the child, for the total number of people living in the child’s household, the participants’ country of origin and understanding what HCPs said about the child’s RSV infection and treatment. Models stratified for the participants’ low /high understanding were adjusted for the same variables except for understanding what HCPs said about the child’s RSV infection and treatment.

Abbreviations: CI, confidence interval; Coef., regression coefficient; HRQoL, health-related quality of life; Ref., reference.

1. **Receiving adequate health information about the child during hospitalisation**

|  | Total | | Parent HRQoL | | Family Functioning | |
| --- | --- | --- | --- | --- | --- | --- |
|  | **Coef. [95% CI]** | **p-value** | **Coef. [95% CI]** | **p-value** | **Coef. [95% CI]** | **p-value** |
| **Overall sample** |  | | | | | |
| Low | Ref. |  | Ref. |  | Ref. |  |
| High | −9.76 [−18.2 to −1.29] | **0.024** | −11.8 [−21.0 to −2.54] | **0.013** | -10.5 [-20.6 to -0.28] | **0.044** |
| **No (adequate) information** |  |  |  |  |  |  |
| Low | Ref. |  | Ref. |  | Ref. |  |
| High | Insufficient data |  | Insufficient data |  | Insufficient data |  |
| **(Adequate) information** |  |  |  |  |  |  |
| Low | Ref. |  | Ref. |  | Ref. |  |
| High | -9.06 [-17.6 to -0.57] | **0.037** | -10.9 [-20.1 to -1.80] | **0.02** | -10.3 [-20.7 to 0.16] | 0.053 |

Models were adjusted for the age, educational level and smoking status of the caregiver, for the current and gestational age of the child, for the total number of people living in the child’s household, the participants’ country of origin and for receiving (adequate) health information about the child during hospitalisation. Models stratified for receiving (adequate) health information about the child during hospitalisation were adjusted for the same variables except for receiving (adequate) information.

Abbreviations: CI, confidence interval; Coef., regression coefficient; HRQoL, health-related quality of life; Ref., reference.

1. **Receiving adequate health information about how to protect the child and family from reinfection**

|  | Total | | Parent HRQoL | | Family Functioning | |
| --- | --- | --- | --- | --- | --- | --- |
|  | **Coef. [95% CI]** | **p-value** | **Coef. [95% CI]** | **p-value** | **Coef. [95% CI]** | **p-value** |
| **Overall sample** |  | | | | | |
| Low | Ref. |  | Ref. |  | Ref. |  |
| High | −8.90 [−17.4 to −0.41] | **0.04** | −10.8 [−20.0 to −1.58] | **0.022** | -9.65 [-20.0 to -0.7] | 0.067 |
| **No information** |  |  |  |  |  |  |
| Low | Ref. |  | Ref. |  | Ref. |  |
| High | -10.3 [-25.6 to 4.91] | 0.177 | -8.62 [-23.7 to 6.42] | 0.252 | -22.4 [-71.7 to 26.8] | 0.33 |
| **Information** |  |  |  |  |  |  |
| Low | Ref. |  | Ref. |  | Ref. |  |
| High | -11.0 [-23.1 to 1.08] | 0.073 | -14.4 [-28.0 to -0.81] | **0.038** | -13.5 [-33.6 to 6.54] | 0.179 |

Models were adjusted for the age, educational level and smoking status of the caregiver, for the current and gestational age of the child, for the total number of people living in the child’s household, the participants’ country of origin and for receiving (adequate) information about how to protect the child and family from reinfection. Models stratified for receiving (adequate) information about how to protect the child and family from reinfection were adjusted for the same variables except for receiving (adequate) information.

Abbreviations: CI, confidence interval; Coef., regression coefficient; HRQoL, health-related quality of life; Ref., reference.

### Table S2: Scale Descriptive Characteristics for PedsQL FIM scores for the subset of participants (n = 59) for whom PedsQL FIM data were obtained on both observation time points: t0 (initial survey) vs. t1 (follow-up survey ~ 6 weeks later)

| **Scale** | **Number of items** | **t0, n = 59**  Mean ± SD | **t1, n = 59**  Mean ± SD | **t(df)** | **p-value^1^** | **ES(d)** |
| --- | --- | --- | --- | --- | --- | --- |
| **Total score** | 36 | 53.98 ± 18.74 | 61.73 ± 21.59 | -4.13 (58) | **< .001** | 0.54 |
| **Parent HRQoL Summary Score** | 20 | 50.47 ± 19.98 | 60.74 ± 21.47 | -5.13 (58) | **< .001** | 0.67 |
| Physical functioning | 6 | 42.2 ± 20.1 | 56.57 ± 21.12 | -5.37 (58) | **< .001** | 0.70 |
| Emotional functioning | 5 | 49.07 ± 24.02 | 63.05 ± 24.23 | -5.64 (58) | **< .001** | 0.73 |
| Social functioning | 4 | 50.95 ± 28.35 | 60.38 ± 28.32 | -2.92 (58) | **0.003** | 0.38 |
| Cognitive functioning | 5 | 61.61 ± 25.06 | 63.73 ± 23.97 | -0.92 (58) | 0.181 | 0.12 |
| Communication | 3 | 67.66 ± 28.87 | 66.1 ± 30.75 | 0.63 (58) | 0.735 | 0.08 |
| Worry | 5 | 51.55 ± 23.55 | 57.88 ± 26.99 | -2.27 (58) | **0.013** | 0.30 |
| **Family functioning Summary Score** | 8 | 59.22 ± 20.38 | 65.15 ± 25.66 | -2.56 (58) | **0.007** | 0.33 |
| Daily activities | 3 | 36.02 ± 28.55 | 53.39 ± 30.45 | -5.22 (58) | **< .001** | 0.68 |
| Family relationships | 5 | 73.14 ± 21.83 | 72.37 ± 26.4 | 0.31 (58) | 0.623 | 0.04 |

t, t-score (paired-samples t); df, degrees of freedom; ES, effect size (Cohen’s d). Total Impact score is computed by averaging all 36 items. HRQOL is computed by averaging the 20 items comprising the Physical, Emotional, Social, and Cognitive Functioning subscales. Family Functioning is computed by averaging the 8 items comprising the Daily Activities and Family Relationships scales. Higher values indicate better HRQOL and family functioning.
